# YTHDF2 promotes spermagonial adhesion through modulating MMPs decay via m^6^A/mRNA pathway

**DOI:** 10.1038/s41419-020-2235-4

**Published:** 2020-01-20

**Authors:** Tao Huang, Zidong Liu, Yi Zheng, Tongying Feng, Qiang Gao, Wenxian Zeng

**Affiliations:** Key Laboratory for Animal Genetics, Breeding and Reproduction of Shaanxi Province, College of Animal Science and Technology, Northwest A&F University, Yangling, Shaanxi 712100 China

**Keywords:** Extracellular matrix, RNA modification, RNAi

## Abstract

As the foundation of male fertility, spermatogenesis is a complicated and highly controlled process. YTHDF2 plays regulatory roles in biological processes through accelerating the degradation of target mRNAs. However, the function of YTHDF2 in spermatogenesis remains elusive. Here, we knocked out *Ythdf2* in mouse spermatogonia via CRISPR/Cas9, and found that depletion of *Ythdf2* mainly downregulated the expression of matrix metallopeptidase (MMPs), thus affecting cell adhesion and proliferation. m^6^A-IP-PCR and RIP-PCR analysis showed that *Mmp3, Mmp13, Adamts1* and *Adamts9* were modified with m^6^A and simultaneously interacted with YTHDF2. Moreover, inhibition of *Mmp13* partially rescued the phenotypes in *Ythdf2*-KO cells. Taken together, YTHDF2 regulates cell-matrix adhesion and proliferation through modulating the expression of *Mmps* by the m^6^A/mRNA degradation pathway.

## Introduction

Male fertility depends on spermatogenesis, which is a complicated and highly controlled process that consists of mitosis of spermatogonia, meiosis of spermatocyte and spermiogenesis^1^. Thus, spermatogonia are the foundation of sperm production^2^. However, the underlying mechanism regulating spermatogonial proliferation remains unclear.

*N*^6^-methyladenosine (m^6^A), the most common internal modification in eukaryotic mRNA, has been shown to participate in mRNA metabolism, including RNA stability and splicing, translation efficiency^3^. Growing evidence indicate that m^6^A is involved in various biological processes, such as cell differentiation, stem cell fate, cardiac remodeling, and cancer progression^4^. m^6^A is a dynamic and reversible modification, which is catalyzed by the methyltransferase complex composed by WTAP, METTL3, METTL14, and other m^6^A “writers”^5^. Meanwhile, this modification can be removed by the m^6^A “eraser” proteins FTO and ALKBH5^6,7^. In addition, m^6^A is recognized by “reader” proteins, including the YTH domain-containing proteins (YTHDF1, YTHDF2, YTHDF3, YTHDC1, and YTHDC2), eIF3 and IGF2BP2^8^.

Recent studies have demonstrated the significance of m^6^A in male fertility. Germ-cell-specific inactivation of METTL3 leads to spermatogenesis arresting at the zygotene stage^9^. Deletion of both *Mettl3* and *Mettl14* in advanced germ cells disrupted spermiogenesis^10^. Knockout of *Alkbh5* caused spermatogonia apoptosis and formation of aberrant sperm^6^. Moreover, Hsu et al. reported that conditional knockout of *Ythdc2* caused germ cells arresting at zygotene stage, thus resulting in male infertility^11^.

YTHDF2 recognizes m^6^A within the GACG motif and mediates degradation of m^6^A-containing transcripts^12^. Until recently, YTHDF2 has been demonstrated to play essential roles in cell processes, such as neural development, cancer progression, maternal mRNAs clearance, and hematopoietic stem cell expansion^13–15^. However, the function of YTHDF2 in male fertility remains elusive. The objective of the present study was to gain more insights into the role of YTHDF2 in spermatogonia proliferation. To this end, we knocked out *Ythdf2* by CRISPR/Cas9 in mouse spermatogonia. We found that depletion of *Ythdf2* affected cell-matrix adhesion and proliferation. We further demonstrated that YTHDF2 mainly regulated the expression of matrix metallopeptidase (MMP) family genes through the m^6^A/mRNA degradation pathway.

## Results

### Depletion of *Ythdf2* via CRISPR/Cas9 in spermatogonia

**Fig. 1:**
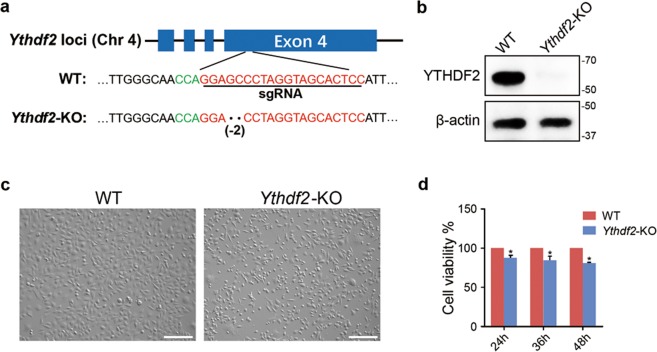
Knockout of *Ythdf2* in mouse spermatogonia cell line. **a** Design of *Ythdf2*-sgRNA and DNA sequence of KO cells. Red letter represents target motif, green letter represents PAM. **b** Detection of YTHDF2 expression in WT cells and *Ythdf2*-KO cells by western blot. *β*-actin was used to eliminate the differences in loading amount. **c** Morphology of WT cells and *Ythdf2*-KO cells. Bar = 50 μm. **d** Cell viability of WT cells and *Ythdf2*-KO cells was detected using CCK-8 assay.

To investigate the function of YTHDF2 in spermatogonia, we designed and synthesized two sgRNAs that targeted the exon 4 of *Ythdf2* loci. SgRNAs were cloned to the PGL-U6 vector. The PGL-sgRNA plasmids and the pST374-Cas9 plasmids were co-transfected to the mouse GC-1 spermatogonial cell line. The cleavage efficiency of the two sgRNAs were detected through the T7E1 assay (Supplementary Fig. 1). Since the sgRNA2 showed a higher cleavage efficiency, we thus picked cell monoclonal from the sgRNA2 transfected cells. Totally, 23 monoclonal cell lines were picked and 11 cell lines were viable. Genotypes of these cell lines were detected through PCR followed by TA-cloning and Sanger sequencing. Among the 11 cell lines, only one cell line showed biallelic frameshift mutation (Fig. 1a), and was regarded as the *Ythdf2*-KO cell line. The depletion of *Ythdf2* was further verified by western blot. As shown in Fig. 1b, expression of YTHDF2 was completely absent in the *Ythdf2*-KO cell line, indicating the successful deletion of *Ythdf2*. The off-target effects were detected via PCR followed by Sanger sequencing. We totally detected 10 of the highest predicted off-target sites and did not observed off-target effects (Supplementary Table 1).

### Depletion of *Ythdf2* decreases cell cycle and cell proliferation

**Fig. 2:**
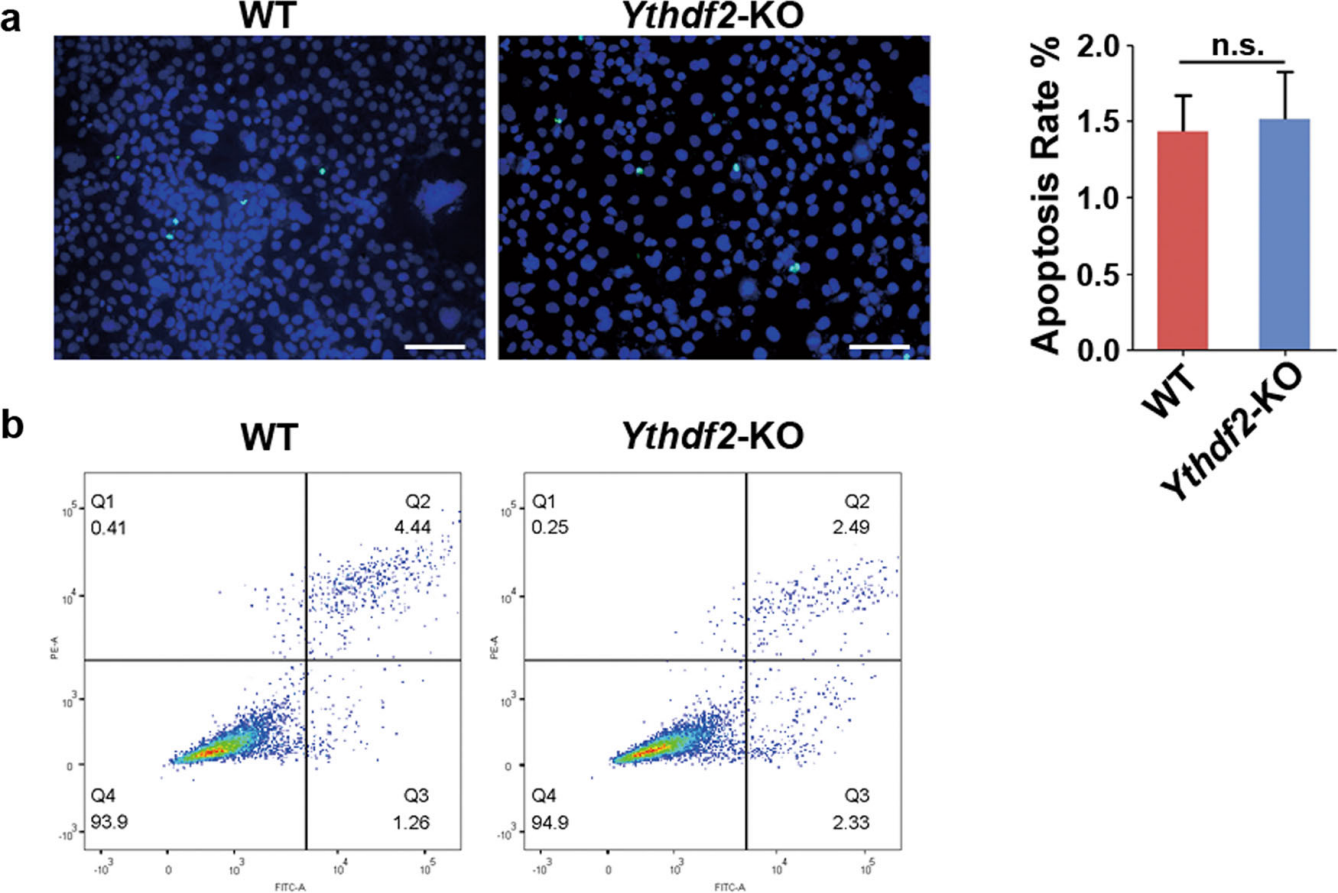
Effects of *Ythdf2*-KO in cell apoptosis. **a** Detection of cell apoptosis in WT cells and *Ythdf2*-KO cells by TUNEL assay. **b** Detection of cell apoptosis in WT cells and *Ythdf2*-KO cells by Annexin V/PI detection kit.

**Fig. 3:**
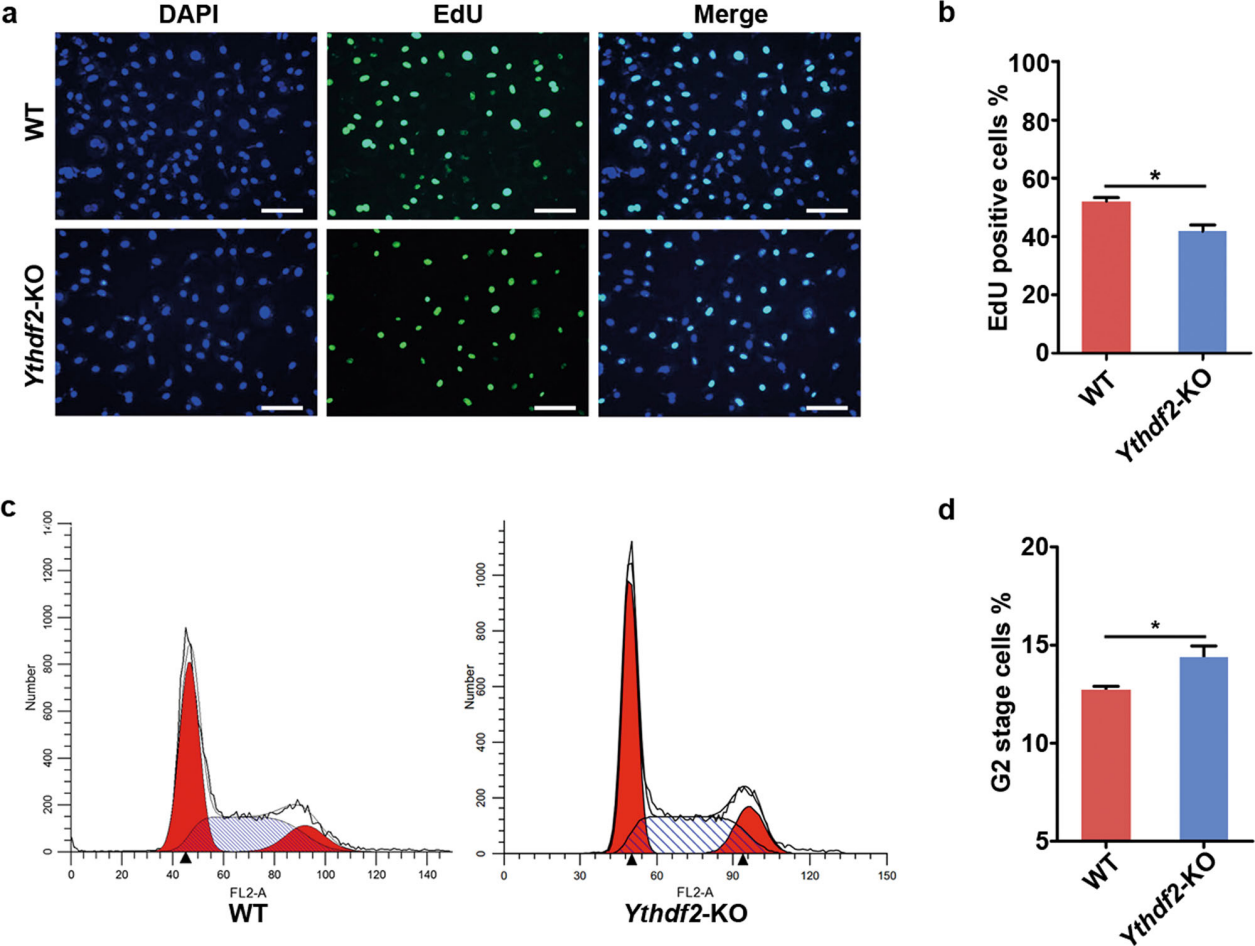
Effects of *Ythdf2*-KO in cell proliferation. **a** EdU staining of WT cells and KO cells. Bar = 50 μm. **b** Analysis of EdU positive cells showed in column diagram. *P < 0.05. **c** Cell cycle analysis of WT cells and KO cells using flow cytometry. **d** Analysis of G2 stage cells showed in column diagram. *P < 0.05.

To disclose the function of YTHDF2 in male germ cells, we first observed the cell morphology and found that the appearance of *Ythdf2*-KO cells changed. Wild type (WT) cells was polygonal, while *Ythdf2*-KO cells were fusiform or round (Fig. 1c). To gain further insights into the function of YTHDF2, we detected the cell viability, and found that cell viability was significantly decreased in *Ythdf2*-KO group (Fig. 1d). TUNEL assay revealed that *Ythdf2*-KO exhibited comparable apoptosis rate with WT (Fig. 2a). We further detected the cell apoptosis via TUNEL combined with Annexin V/PI co-staining assay. Consistently, we found that depletion of YTHDF2 did not affect cell apoptosis (Fig. 2b). But, EdU assay showed that the EdU positive rate in *Ythdf2*-KO group was significantly lower than that in WT group, indicating that knockout of *Ythdf2* inhibited spermatogonial proliferation (Fig. 3a, b). Flow cytometry analysis demonstrated that *Ythdf2*-KO led to significant increase in the rate of cells at G2 stage, indicating that depletion of *Ythdf2* affected G2/M transition (Fig. 3c, d).

### YTHDF2 affects cell adhesion and cell spread

**Fig. 4:**
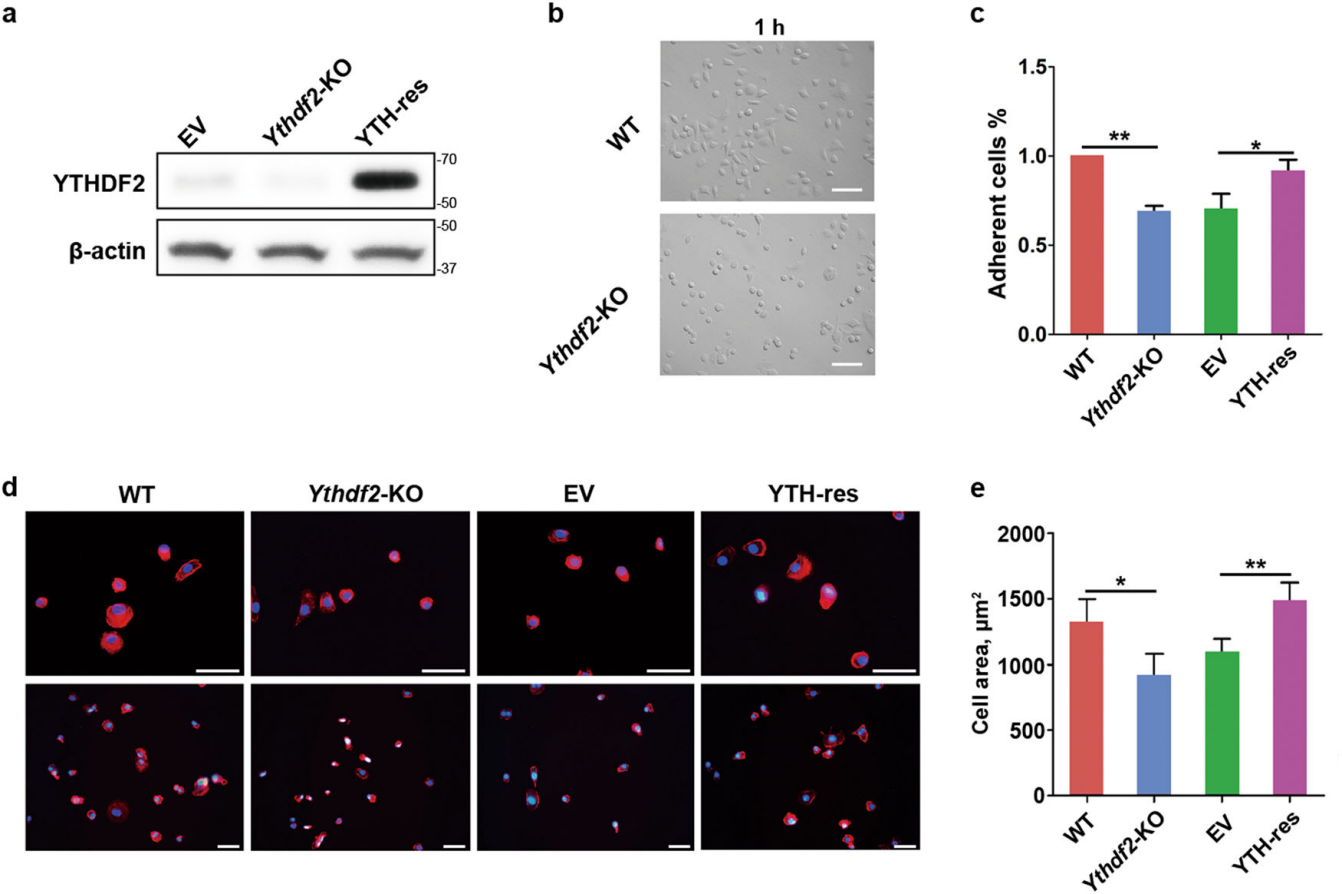
Effects of *Ythdf2*-KO on cell adhesion and cell spread. **a** Detection of YTHDF2 expression by western blot. YTH-res: YTHDF2-rescue cell line. EV empty-vector control. **b** Morphology of cells adhered for 1 h. Bar = 50 μm. **c** Detection of adherent cells of each group by CCK-8 assay. *P < 0.05, **P < 0.01. **d** Detection of cell spreading area by F-actin staining. Bar = 50 μm. **e** Analysis of cell area showed in column diagram. *P < 0.05, **P < 0.01.

Since the cell morphology was changed by *Ythdf2*-KO, to further investigate the regulatory role of YTHDF2 in the morphology transition, we detected the cell-adhesion ability. Interestingly, the rate of adherent cells in *Ythdf2*-KO cells was significantly lower than WT group, indicating that depletion of *Ythdf2* decreased cell adhesion (Fig. 4b, c). Since previous studies reported that the circularity of adherent cells was associated with cell spread, we thus detected the cell spread. Cells were stained with FITC-labeled phalloidin and 4’,6-diamidino-2-phenylindole (DAPI). We found that the average cell spread area in *Ythdf2*-KO group was significantly smaller than WT group, indicating that knockout of *Ythdf2* decreased cell spread (Fig. 4d, e).

To exclude the off-target effects on phenotypes, we established the YTHDF2-rescue cell line (YTH-res) that stably recovered YTHDF2 in the *Ythdf2*-KO cells. The expression of YTHDF2 in the YTH-res cells were verified by western blot (Fig. 4a). We next detected the phenotypes of YTH-res cells and compared with the empty-vector control (EV). The phenotypes in cell adhesion and cell spread could be partially rescued in YTH-res group (Fig. 4c–e), indicating that YTHDF2 regulates cell adhesion and cell spread.

### YTHDF2 affects the expression of extracellular matrix

**Fig. 5:**
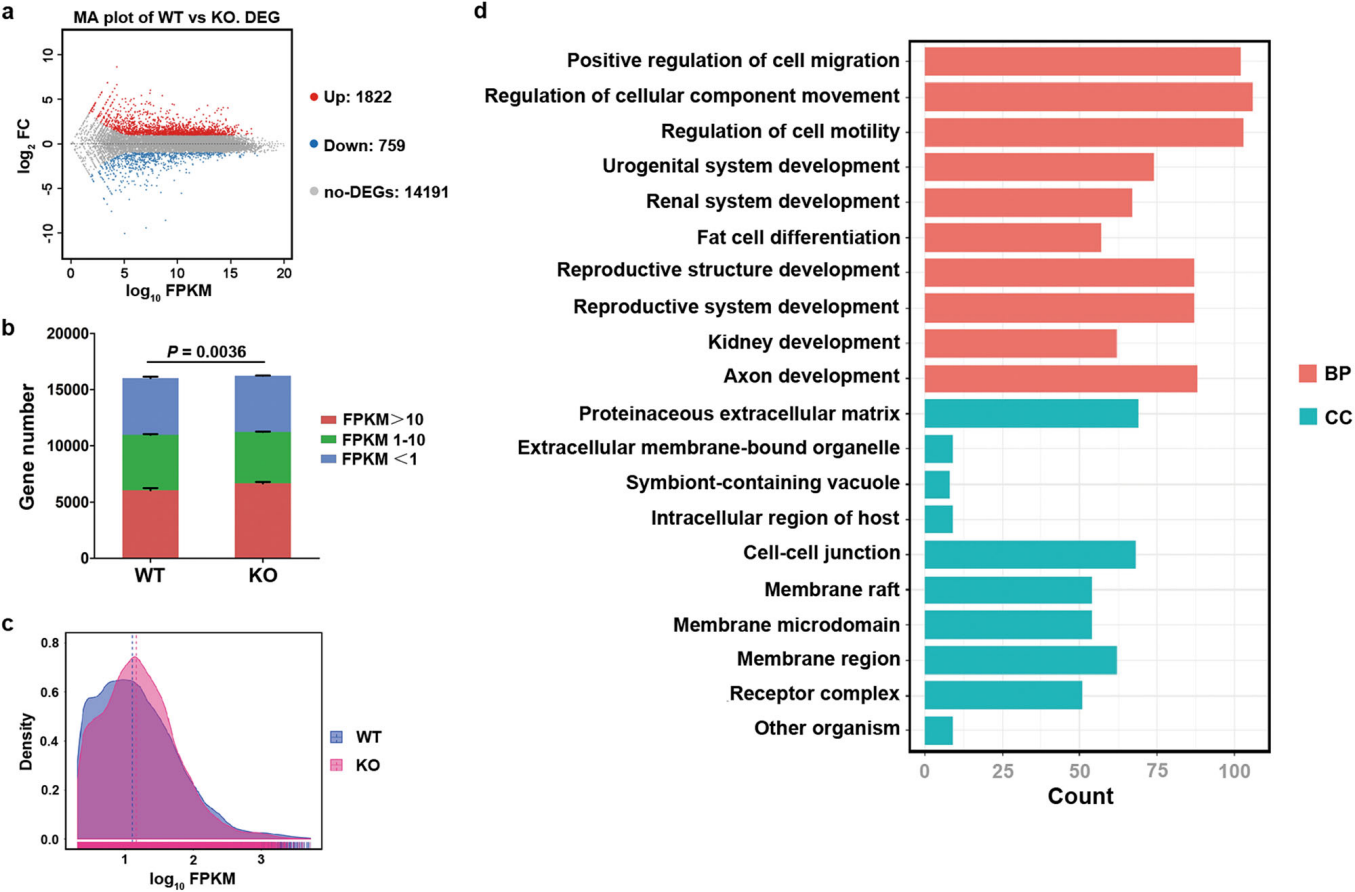
RNA-seq analysis of WT cells and *Ythdf2*-KO cells. **a** MA plot analysis showed the number of differentiated expressed genes (DEGs). Up: upregulated expressed genes. Down: downregulated expressed genes. **b** Count of genes with different FPKM value showed in column diagram. **c** Analysis of gene expression density in WT cells and KO cells. **d** GO analysis of DEGs between WT cells and KO cells. GO terms were ranked by adjust P-value. BP: biological processes. CC cell components.

To reveal the underlying mechanisms, we performed RNA-seq. By comparing the transcriptome between *Ythdf2*-KO cells and WT cells, we identified 2581 genes that were differentially expressed ≥ 2-fold, of which 1822 were upregulated and 759 were downregulated in *Ythdf2*-KO cells (Fig. 5a). Density plots showed that the global expression level of transcriptome was enhanced by *Ythdf2* depletion (Fig. 5b, c).

**Fig. 6:**
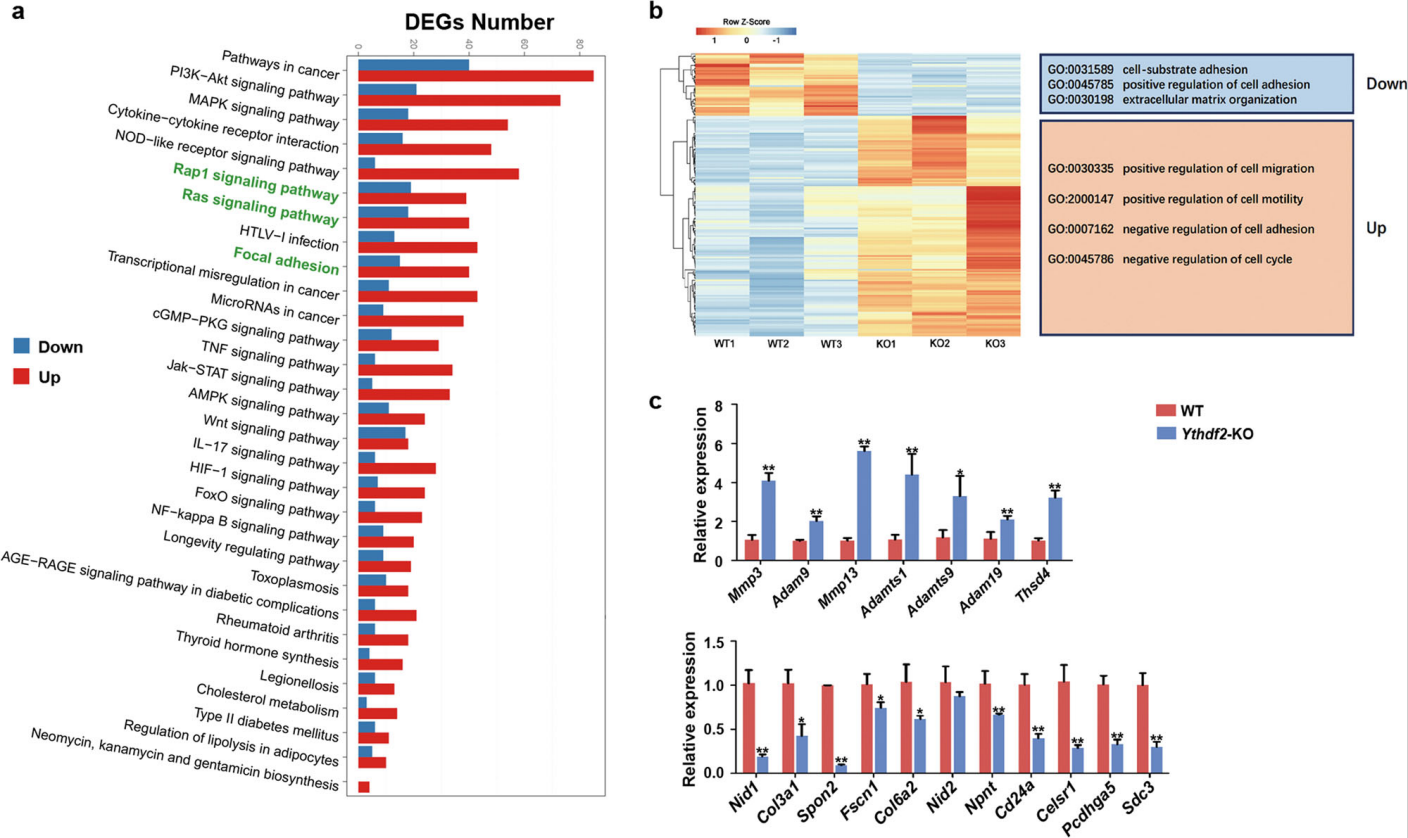
Analysis and verification of DEGs between WT cells and *Ythdf2*-KO cells. **a** KEGG analysis of DEGs. Terms in green typeface mean phenotype relative pathways. **b** Heatmap analysis of DEGs associated with phenotypes. **c** Expression of the targeted genes was verified by RT-qPCR. *P < 0.05, **P < 0.01.

Gene ontology (GO) analysis of the differentiated expressed genes (DEGs) was annotated by biological processes (BP) and cellular components (CC), respectively. As shown in Fig. 5d, GO-BP analysis showed that the DEGs were mainly involved in cell migration-associated processes including positive regulation of cell migration, regulation of cellular component movement and regulation of cell motility. GO-CC analysis showed that the DEGs were predominantly linked to extracellular components including proteinaceous extracellular matrix, extracellular membrane-bound organelle, and symbiont-containing vacuole. We next focused on the genes involved in the GO terms directly associated with cell adhesion. We found that the downregulated genes were mainly linked to processes that promoting cell-matrix adhesion and extracellular matrix organization. By contrast, the upregulated genes were principally involved in the processes promoting cell migration and motility, inhibiting cell adhesion, and negative regulation of cell cycle (Fig. 6b). Kyoto Encyclopedia of Genes and Genomes (KEGG) pathway analysis showed that DEGs were enriched in pathways in cancer, PI3K-Akt signaling pathway and MAPK pathway. Noticeably, Rap1 signaling pathway, Ras signaling pathway and focal adhesion pathway, which associated with cell-matrix adhesion, were ranked at the forefront (Fig. 6a).

We next focused on the DEGs in cell adhesion associated GO terms. Genes that were highly expressed and showed significant difference were selected as target genes. *Mmp3, Adam9, Mmp13, Adamts1, Adamts9, Adam19*, and *Thsd4* were the upregulated genes, which were mainly belonged to the matrix metalloproteinase (MMP) family. *Nid1, Col3a1, Spon2, Fscn1, Col6a2, Nid2, Npnt, Cd24a, Celsr1, Pcdhga5*, and *Sdc3* were the downregulated genes, which were mainly belonged to the extracellular matrix (ECM). q-PCR analysis further verified the RNA-seq data (Fig. 6c). Taken together, depletion of *Ythdf2* affected cell-matrix adhesion mainly through modulating the expression of the MMPs and ECMs.

### YTHDF2 regulates the degradation of m^6^A modified MMP mRNAs

**Fig. 7:**
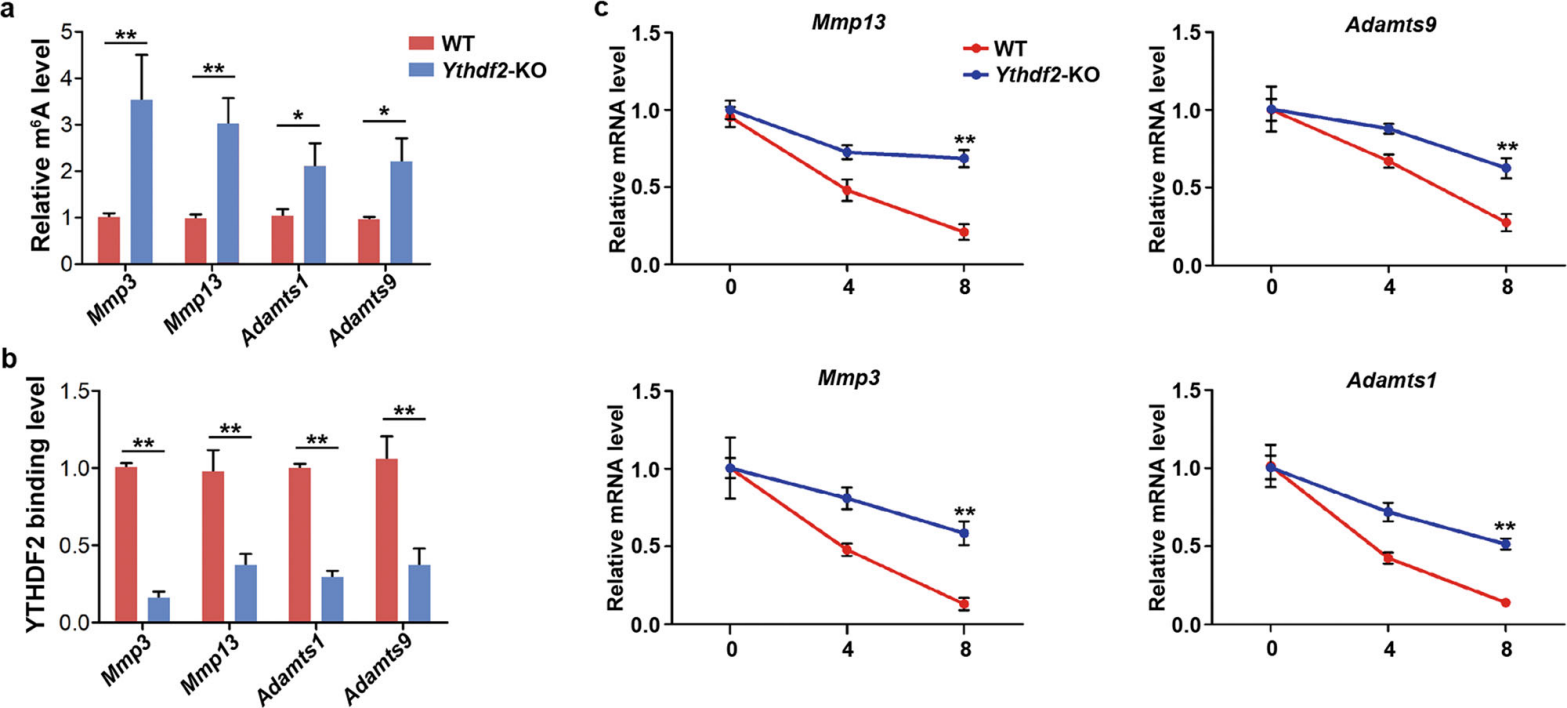
Verification of the regulation of YTHDF2 on the targeted genes. **a** m6A level of targeted genes was detected using m6A-IP-qPCR. *P < 0.05, **P < 0.01. **b** Verification of the interaction between YTHDF2 and targeted mRNAs by RIP-qPCR. *P < 0.05, **P < 0.01. **c** Detection of the mRNA stability of target genes using RNA-decay assay.

RNA-seq analysis showed that changes in the expression of ECMs and MMPs mainly contributed to cell adhesion. Previous studies have reported the acceleration of YTHDF2 on the degradation of m^6^A modified mRNAs. Hence, we hypothesized that genes whose expression were upregulated by *Ythdf2* depletion, were the targets of YTHDF2. To this end, we performed m^6^A-IP-PCR to verify the m^6^A modification on the targeted genes. *Mmp3, Mmp13, Adamts1*, and *Adamts9*, which were associated with the phenotypes and contained the top *P*-values analyzed by RNA-seq, were subjected to m^6^A-IP-PCR. As shown in Fig. 7a, the relative m^6^A level of these four targeted genes was significant higher in *Ythdf2*-KO cells than that in WT cells, indicating that these targeted mRNAs were modified with m^6^A.

To verify the interactions between these targeted transcripts and YTHDF2, we performed RIP-PCR using YTHDF2 antibody. As shown in Fig. 7b, the relative binding level of the targeted mRNAs in *Ythdf2*-KO cells were significantly higher than that in WT cells, indicating the interactions between YTHDF2 and the targeted transcripts. To elaborate whether YTHDF2 accelerated the degradation of the targeted mRNAs, we performed RNA-decay assay as previously described. We found that at 8 h post-treatment, the residual amount of the targeted mRNAs in *Ythdf2*-KO group was significantly higher than that in WT group (Fig. 7c), indicating that YTHDF2 promoted the degradation of targeted mRNAs. Thus, YTHDF2 regulated the expression of MMPs through modulating the degradation of m^6^A modified MMPs mRNAs.

### Knockdown of *Mmp13* rescues the phenotypes induced by YTHDF2 KO

**Fig. 8:**
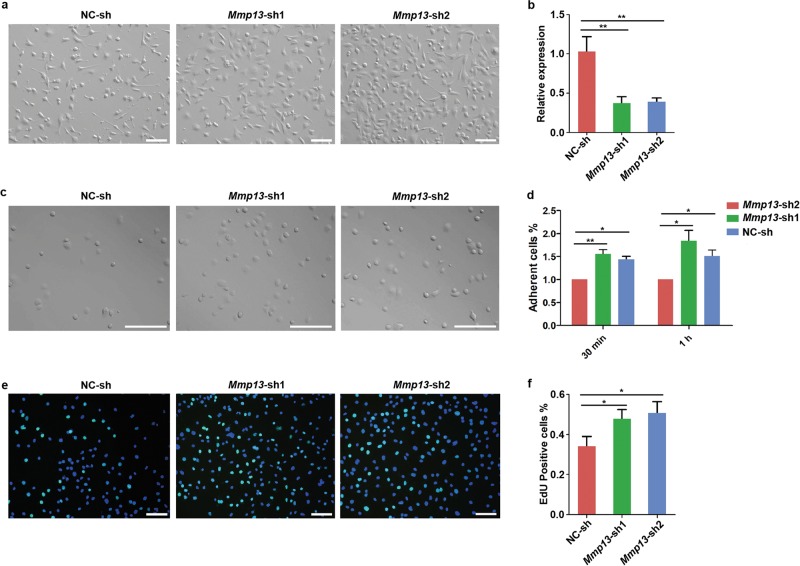
Rescue of the *Ythdf2*-KO Phenotypes by interfering *Mmp13*. **a** Morphology of *Mmp13*-sh cells and the negative control. **b** Knockdown efficiency of *Mmp13* shRNAs was detected by q-PCR. **c** EdU staining of control cells and *Mmp13*-sh cells. **d** Analysis of EdU positive cells showed in column diagram. **e** Morphology of adherent cells. Bar = 50 μm **f** Relative cell adherent ability was detected by CCK-8 assay. *P < 0.05, **P < 0.01.

The MMPs are well-studied enzymes that mediate the degradation of various extracellular matrixes. Among the verified target genes, *Mmp13* contained the lowest *P* value analyzed by RNA-seq, which means that it was relatively high expressed and showed larger differences. We therefore hypothesized that the *Mmp13* may plays important roles in the regulation of cell adhesion and proliferation. To verify the hypothesis, we knockdown the expression of *Mmp13* in *Ythdf2*-KO cells using RNAi mediated by lentivirus. The interferential efficiency was measured by q-PCR (Fig. 8a, b). Cell-matrix adhesion ability of *Mmp13* knockdown by shRNA (*Mmp13*-sh) and shRNA negative control (NC-sh) cells were detected through cell-adhesion assay. Notably, *Mmp13*-sh cells showed significantly higher adhesion ability compared with NC-sh cells (Fig. 8c, d). Additionally, cell proliferation rate in *Mmp13*-sh group was significantly higher than that in NC-sh group (Fig. 8e, f). These results indicated that inhibition of MMP13 partially rescued the phenotypes induced by *Ythdf2*-KO. Taken together, the regulation of YTHDF2 on cell-matrix adhesion and proliferation was through, at least partially, modulating the expression of *Mmp13*.

## Discussion

m^6^A modification have been demonstrated to be essential for spermatogenesis. *Mettl3* or *Mettl14* deficiency induced the abnormal initiation of spermatogonial differentiation, and spermatocytes are unable to reach the pachytene stage of meiotic prophase^10^. In addition, *Alkbh5* deficiency results in aberrant splicing and generation of shorter transcripts in the spermatocytes and round spermatids^6^. Immortalized germ-cell lines were wildly used for studying regulatory mechanism of spermatogenesis, such as C18-4 cell line (type A spermatogonia with stemness), GC-2 cell line (primary spermatocytes), GC-4spc cell line (the stage between preleptotene and early pachytene spermatocytes)^16–18^. To detected the detailed roles of YTHDF2 in transition of spermatogonia to spermatocytes, GC-1 spermatogonial cell line, a stage between type B spermatogonia and primary spermatocytes^19^, were used for further analysis. Here, we found that depletion of *Ythdf2* suppressed cell-matrix adhesion and cell cycle in spermatogonia, and that *Mmp13* was an important target of YTHDF2 in the regulation of phenotypes.

The association between m^6^A and the migration of various cancer cells has been well reported. In bladder cancer cells, METTL3 and ALKBH5 alter cell adhesion through the m^6^A methylations of the ITGA6 mRNA 3’UTR^20^. Interfering of YTHDF2 upregulates the expression of MMP2 and MMP9 that possess the capability to promote cell adhesion^21,22^, thus enhancing the invasion and adhesion of pancreatic cancer cells^23^. However, in the present study, we found that depletion of *Ythdf2* led to a decrease in the cell-matrix adhesion. In the *Ythdf2*-KO cells, the expression of various extracellular matrixes was changed. Contrasted to that in pancreatic cancer cells, the expression of *Mmp2* did not change, and *Mmp9* was downregulated by *Ythdf2*-KO in spermatogonia. This may explain the contradictory between spermatogonia and pancreatic cancer cells.

Cell-matrix adhesion is indispensable for cell migration, cell differentiation^24–26^. In male reproductive system, cell–cell adhesion and cell-matrix adhesion are important for the differentiation of germ cells^27,28^. In the seminiferous tubules, spermatogonia differentiate into preleptotene spermatocytes that are within the basal compartment, and the latter migrate from the basal to the adluminal compartment. Spermatogonia and preleptotene spermatocytes are bound tightly to neighboring Sertoli cells by desmosome-like junctions^29^. Thus, the cell-adhesion molecules located at the surface of spermatogonia are essential for differentiation and migration^30,31^. In the present study, depletion of *Ythdf2* affected the expression of ECM genes and MMP genes. Notably, most ECM genes were downregulated, whereas MMP genes were upregulated. Among the upregulated MMP genes, *Mmp13, Mmp10, Adamts1*, and *Adamts9* were highly expressed in spermatogonia. We then verified that m^6^A occurred in these YTHDF2 targeted genes. It has been demonstrated that YTHDF2 can accelerate the degradation of m^6^A modified mRNAs^32^. In the present study, depletion of *Ythdf2* significantly enhanced the stability of the targeted mRNAs, which were consistent with previous studies^32^. Additionally, knockdown of MMP13 by shRNA in *Ythdf2*-KO cells partially restored the phenotypes, indicating that *Mmp13* is a downstream signal of YTHDF2 in the regulation of cell-matrix adhesion. It is no doubt that the influence of YTHDF2 is global, thus the regulation of YTHDF2 on cell-matrix adhesion may not only through *Mmp13*, but also through other pathways such as Rho signaling or Ras signaling.

The matrix metalloproteinases, which are known to be responsible for the degradation of ECM, are reported to be associated with spermatogenesis, cell junctions, and sperm-egg binding^30^. Importantly, MMP13 acts as a central factor in the MMP activation cascade^33,34^. Previous studies have reported the regulatory role of MMP13 in bone metabolism and the invasion and metastasis of tumor^35^. MMP13 has the ability to cleave various types of collagens (I, II, III, and IV), gelatin, aggrecan, and perlecan^34^. Association between MMP13 and the metastasis of tumors such as breast carcinoma^36^, skin carcinoma^37,38^, and lung cancer^39^, has been wildly revealed. Here, we showed that the m^6^A modification occurred in *Mmp13* was regulated by YTHDF2, and that MMP13 participated the regulation of cell-matrix adhesion in spermatogonia.

In conclusion, YTHDF2 regulates spermatogonia migration and proliferation, at least partially, through affecting the stability of m^6^A modified MMPs genes. Since the present study was based on the in vitro cell model without the precise regulation of somatic cell populations in testis, such as Leydig cells, peritubular myoid cells, and Sertoli cells. To gain further insights into the regulatory role of YTHDF2 on male fertility, it is optimal to establish germ-cell conditional *Ythdf2*-KO mouse.

## Materials and methods

### Cell culture and plasmids transfection

Mouse spermatogonia (GC-1 cells, a gift from Shanghai Institute for Biochemistry and Cell biology) were cultured in Dulbecco’s Modified Eagle’s Medium (DMEM, Hyclon) with 10% fetal bovine serum (FBS, Gibico), 100 U/mL penicillin and 0.1 mg/mL streptomycin (PS) and incubated at 37 °C with 5% CO2. The 293 T cells were cultured in DMEM with 10% FBS, 6 mmol/L L-Glutamine, 0.1 M MEM NEAA and 1 × Penicillin-Streptomycin. Plasmids were transfected to cells using the TurboFect^TM^ Transfection Reagent (Thermo Scientific^TM^) following the manufacturer’s instructions.

### Plasmids construction

To knockout *Ythdf2* in spermatogonia, the following sgRNAs were synthesized. sgYTHDF2U: 5’-ACCGGAGTGCTACCTAGGGCTCC-3’, sgYTHDF2D: 5’- AAACGGAGCCCTAGGTAGCACTC-3’. The PGL3-U6-PGK plasmid (gifted from Shanghai Tech University) was digested with Bsa I and purified using the PCR clean-up kit (Axgen). The up and down sgRNAs were annealed and ligated with PGL3-U6-PGK using T4 ligase (Thermo). For the YTHDF2-rescue experiment, the following primers were synthesized, YTHres-F: 5’-CAGGAATTCATGTCGGCCAGCAGCCTCTT-3’, YTHres-R: 5’-CGAGGATCCCTATTTCCCACGACCTTGACG-3’. Total RNA of spermatogonia was extracted using RNAiso plus Reagent (Takara Clonetech) following the manufactures’ instructions. cDNA was synthesized using the Transcriptor First Strand cDNA Synthesis Kit (Rocher). Complete CDS of *Ythdf2* was amplified by PCR and purified using the Gel extraction kit (TianGen) followed by ligated with the CD513B-CMV plasmids (gift from Dr. Enqi Du, Northwest A&F university).

### Establishment of *Ythdf2*-KO cells

Spermatogonia was cultured to 50% confluence. Plasmids expressing Cas9 and sgRNAs were co-transfected to the cells using the TurboFect^TM^ transfection Reagent (Thermo) following the manufactures’ instructions. Twenty-four hours post-transfection, cells were subjected to drug screen using 2 μg/ml puromycin (Sigma) for 48 h. A portion of living cells were collected and subjected to DNA extraction using the QuickExtract DNA Extraction Solution 1.0 (Epicenter). The efficiency of indels was detected by T7E1 assay using the T7 endonuclease (NEB). Residual cells were seeded to the 100-mm-dish at the density of 3000 cells per dish.

### Lentivirus package

Two hundred and ninety three T cells were cultured in Dulbecco’s Modified Eagle’s Medium (DMEM, Hyclon) with 10% fetal bovine serum, glutamine (Gibco), nonessential amino acid (NEAA, Gibco), 100 U/ml penicillin and 0.1 mg/ml streptomycin (PS). For lentivirus package, cells were cultured to 90% confluence. Plasmids were co-transfected to cells. Cell suspension was removed at 16 h post-transfection.

### m^6^A dot blot

Total RNA was extracted from the cells using Trizol reagent (TAKARA). mRNA was isolated and purified using Poly Attract mRNA Isolation System III with Magenetic Stand (Promega) following the manufacturer’s instructions. For m^6^A dot blot, mRNA was hybridized onto the Hybond-N + membrane (GE Healthcare). After crosslinking at 80 °C for 30 min, the membrane was blocked with 5% non-fat milk (Biorad) for 1 h, incubated with rabbit anti-m^6^A antibody (1:1000, Synaptic Systems, cat. No. 202003) at 4 °C overnight. Then the membrane was incubated with HRP-conjugated mouse anti-rabbit IgG (1:3000, Santa,sc-2357) at room temperature for 2 h. After being incubated with Immobilon Western Chemiluminescent HRP Substrate (Millipore), the immunocomplex was photographed using the ECL imaging system (Bio-Rad). Finally, the membrane was stained with 0.02% methylene blue to eliminate the difference in mRNA amount. Relative m^6^A level was quantified via gray intensity analysis using Image J.

### Western blot assay

Cells were lysed with RIPA buffer containing 1% PMSF followed by ultrasonication. Cell lysates were incubated on ice for 30 min, centrifuged at 10,000 × *g* for 10 min. The supernatants were collected and the protein concentration was detected using a BCA detection Kit. Equal amount of proteins was loaded to the polyacrylamide gel. The proteins were separated through SDS-PAGE using the electrophoresis apparatus (Bio-Rad). After electrophoresis, the proteins were transferred to the PVDF membrane (Millipore, IBFP0785C) using a semi-dry transfer instrument (Bio-Rad). The membranes were blocked with 5% non-fat milk for 1 h at room temperature, incubated with primary antibodies at 4 °C overnight. Subsequently, the membranes were washed with PBST and incubated with HRP-conjugated secondary antibodies for 1 h at room temperature. After washing, the membranes were incubated with the Immobilon Western Chemiluminescent HRP Substrate (Millipore, USA) and photographed using the ECL imaging system (Bio-Rad, USA).

### Flow cytometric analysis

For cell cycle analysis, the cells were suspended in 75% cold ethanol and treated with 0.1% Triton X-100 and 100 μg/ml RNase at 37 °C for 30 min. Subsequently, the cells were stained with 50 μg/ml PI for 2 h and analyzed by flow cytometry. For cell clustering analysis, cells were fixed in cold 70% ethanol, permeablized with 0.1% Triton X-100. Then the cells were stained with 4’,6-diamidino-2-phenylindole (DAPI, Thermo) for 30 minutes and analyzed by flow cytometry.

### RNA-decay assay

WT and *Ythdf2*-KO cells were treated with 5 μg/mL actinomycin D for 0, 3, and 6 h. Cells were harvested at each time point and subjected to RNA extraction. Real-time quantitative PCR were used to analyze the mRNA level of target genes in each group.

### Cell-adhesion assay

Cells were tryosinized, resuspended with complete culture medium, and seeded at 1 × 10^4^ on 96-well plates that were pre-coated with 10 μg/ml fibronectin (Sigma–Aldrich) at 37 °C for 2 h. After 30 min incubation, culture medium was removed and the nonspecific adherent cells were washed out with PBS for three times. Residual cells were counted using CCK-8 assay (Beyotime).

### MeRIP-PCR

MeRIP-PCR was performed following the previous study^40^. Briefly, total RNA was extracted from 1 × 10^7^ cells using TRIZOL (Thermo). mRNA was isolated using the PolyATtract^®^ mRNA Isolation Systems (Promega, Z5310) following the manufacturer’s instructions. IP mixture was composed by 6 μg rabbit anti m^6^A antibody (Synaptic System, cat. No. 202003), 3 μg mRNA, IP buffer (50 mmol/L Tris-HCl, pH 7.4, 750 mmol/L NaCl, and 0.5% NP-40), RNA inhibitor (Thermo) and RNase-free water up to 500 μL in total volume. After being mixed by rotating for 2 h at 4 °C, the IP mixture was incubated with the protein A beads which have been washed for three times and blocked by 0.5 mg/mL BSA, followed by rotating overnight at 4 °C. Precipitated mRNA was eluted using elution buffer (1 × IP buffer, 6.7 mM m^6^A). For the detection of relative m^6^A level, 40 ng of precipitated mRNA and input RNA was subjected to cDNA synthesis and quantitative PCR, respectively.

### Quantitative real-time PCR

Cells were lysed with Trizol regent (TAKARA). Total RNA was isolated by chloroform followed by precipitating with isopropanol. cDNA was synthesized with the PrimeScript™ RT reagent Kit (TAKARA) following the manufactory’s instructions. Primers designed and synthesized for RT-qPCR were listed in Supplementary Table 1. Quantitative PCR was performed using the SYBR Green II PCR Mix (TAKARA) and the IQ5 (Bio-Rad).

### Statistical analysis

All data were collected from at least three independent experiments. Data were analyzed using two-tailed student’s *t*-test or one-way ANOVA followed by a Duncan’s multiple range test (SPSS 22 for windows). Significance were presented as *p < 0.05, **p < 0.01, and ***p < 0.001. Error bars represented SEM of the mean.

## Supplementary information

Fig. S1

Fig. S2

Supplemementary Figure Legends

YTHDF2-sgRNA2 off targets
