# Supplementary figures and images for: YTHDF2 promotes spermagonial adhesion through modulating MMPs decay via m6A/mRNA pathway
Source: Cell Death Dis. 2020 Jan 20;11(1):37. doi: 10.1038/s41419-020-2235-4 (PMC6971064; doi:10.1038/s41419-020-2235-4)

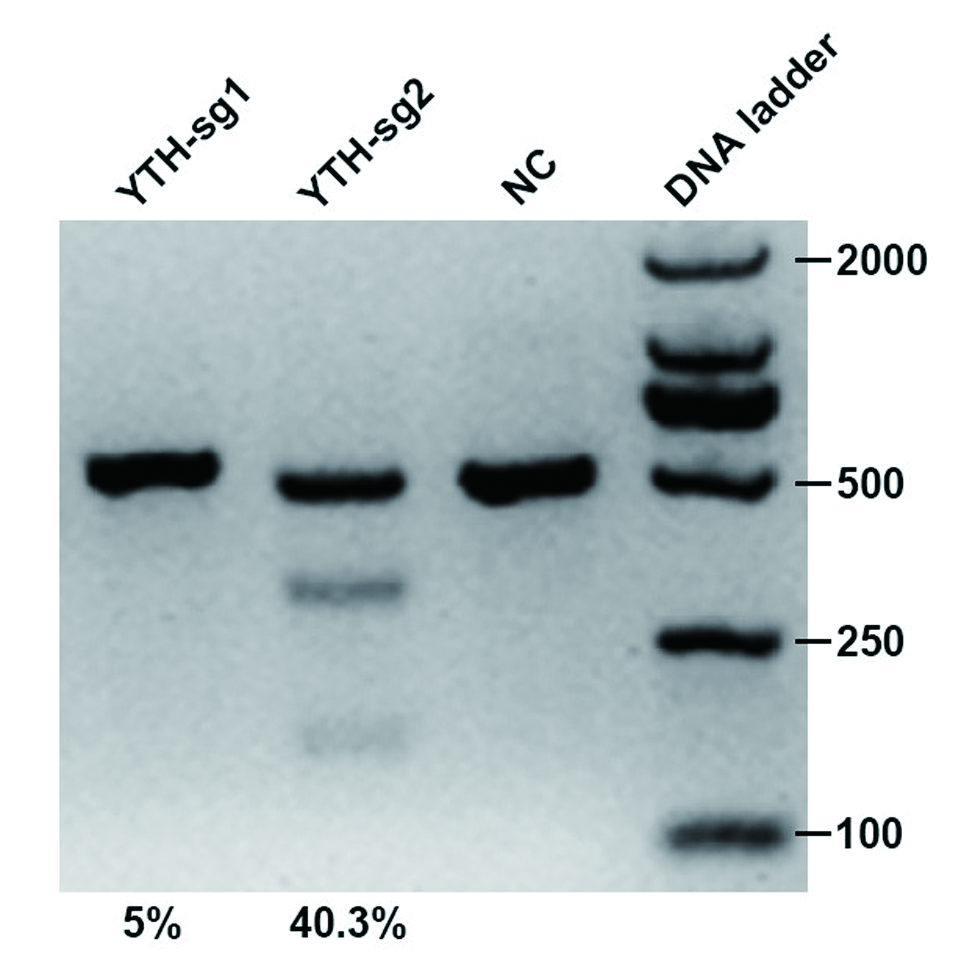

Supplement: Supplementary file 1 — Fig. S1 [file 41419_2020_2235_MOESM1_ESM.tif]

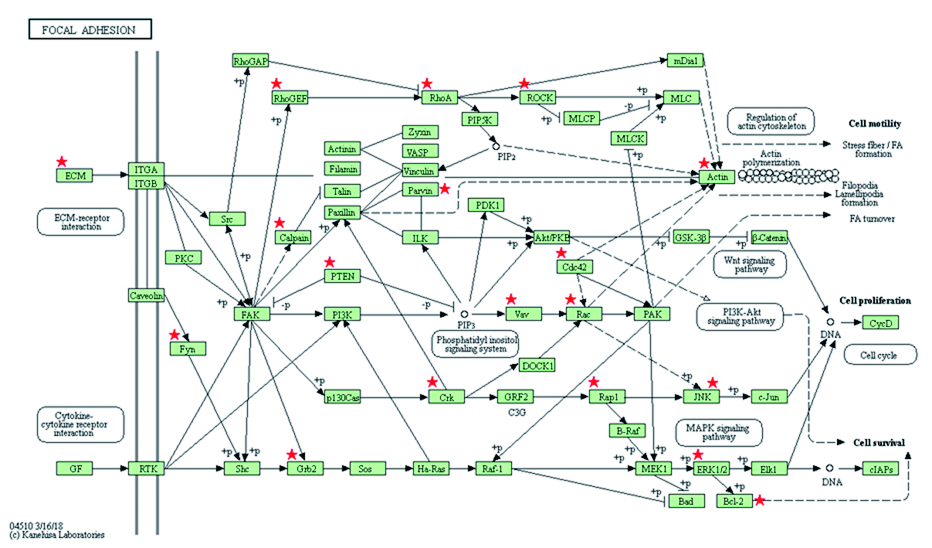

Supplement: Supplementary file 2 — Fig. S2 [file 41419_2020_2235_MOESM2_ESM.tif]
