# Supplementary material for: YTHDF2 promotes spermagonial adhesion through modulating MMPs decay via m6A/mRNA pathway
Source: Cell Death Dis. 2020 Jan 20;11(1):37. doi: 10.1038/s41419-020-2235-4 (PMC6971064; doi:10.1038/s41419-020-2235-4)
Supplement: Supplementary file 3 — Supplemementary Figure Legends [file 41419_2020_2235_MOESM3_ESM.docx]

**Figure S1.** Cleavage efficiency of sgRNAs detected by T7E1 assay. 200 ng of DNA was loaded and subjected for electrophoresis for 1 hour. The cleavage efficiency was calculated by grayscale analysis.

**Figure S2**. Focal adhesion pathway refered to the KEGG database. Differentiated expressed genes were marked by red star.
